# Cyclodextrin Complexes for Clinical Translatability: Applications for Cladribine and Retrometabolically Designed Estredox

**DOI:** 10.3390/ijms262210976

**Published:** 2025-11-13

**Authors:** Nicholas Bodor, Peter Buchwald

**Affiliations:** 1 Bodor Laboratories, Miami, FL 33137, USA; nsbodor@bodorlabs.com; 2 Diabetes Research Institute, Miller School of Medicine, University of Miami, Miami, FL 33136, USA; 3 Department of Molecular and Cellular Pharmacology, Miller School of Medicine, University of Miami, Miami, FL 33136, USA

**Keywords:** blood–brain barrier, brain targeting, complexation energy, dual complex, estradiol, oral bioavailability, multiple sclerosis, solubility, physicochemical properties, prodrug

## Abstract

In this study, we review the use of cyclodextrin-based formulations to develop oral tablets of cladribine by enhancing its bioavailability and to improve the solubility and stability of retrometabolic chemical delivery systems (CDSs) in general and estredox, a brain-targeting estradiol-CDS, in particular. Cyclodextrins (CDs), cyclic oligosaccharides that can form host–guest inclusion complexes with a variety of molecules, are widely utilized in pharmaceuticals to increase drug solubility, stability, bioavailability, etc. The stability of the complex depends on how well the guest fits within the cavity of the CD host; a model connecting this to the size of the guest molecules is briefly discussed. Modified CDs, and particularly 2-hydroxypropyl-β-cyclodextrin (HPβCD), provided dramatically increased water solubility and oxidative stability for estredox (estradiol-CDS, E_2_-CDS), making its clinical development possible and highlighting the potential of our brain-targeted CDS approach for CNS-targeted delivery with minimal peripheral exposure. A unique HPβCD-based formulation also provided an innovative solution for the development of orally administrable cladribine. The corresponding complex dual CD-complex formed by an amorphous admixture of inclusion- and non-inclusion cladribine–HPβCD complexes led to the development of tablets that provide adequate oral bioavailability for cladribine, as demonstrated in both preclinical and clinical studies. Cladribine–HPβCD tablets (Mavenclad) offer a convenient, effective, and well-tolerated oral therapy for multiple sclerosis, achieving worldwide approval and significant clinical success. Overall, the developments summarized here underscore the importance of tailored cyclodextrin-based approaches for overcoming barriers in drug formulation for compounds with challenging physicochemical properties, and demonstrate the versatility and clinical impact of CD inclusion complexes in modern pharmaceutical development.

## 1. Introduction

**Figure 1:**
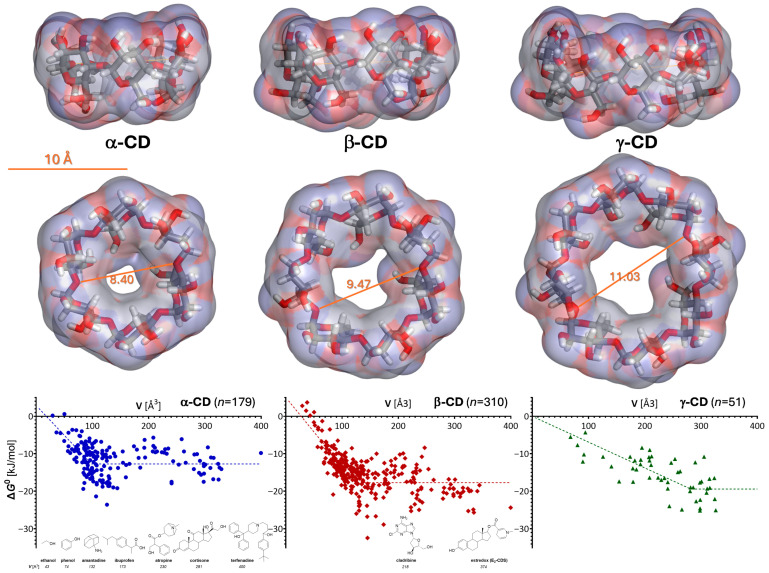
Cyclodextrin structures and their host–guest complexation energies. Optimized three-dimensional structures of the natural α-, β-, and γ-cyclodextrins are shown at the top as stick structures covered by a semitransparent soft surface colored by atomic charge from two different angles with a scale bar included. The dependence of the corresponding complexation energies for 1:1 complexes is shown at the bottom as a function of the molecular size (volume, *V*) of the guest [8,9]. Symbols are standard free energies of 1:1 complexation (Δ*G*^0^) for *n* different guests, as indicated; the dotted lines represent the best fit obtained with a bilinear segment (see text for details). Some representative chemical structures for selected guest molecules with experimentally determined complexation energies, as well as estredox and cladribine, which are discussed here, are shown at the bottom, positioned according to their molecular volume *V* to illustrate the size scale.

**Figure 2:**
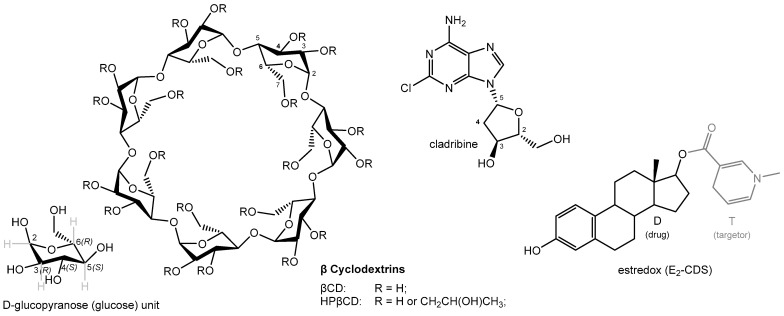
Chemical structures of the molecules discussed here, including βCD, HPβCD, cladribine, and estredox (E_2_-CDS). A glucose (D-glucopyranose) building block unit of CDs is shown in the bottom left corner, with stereochemical details for illustration.

Cyclodextrins (CDs) represent a family of torus-shaped cyclic oligosaccharides, which can be enzymatically derived from starch [1,2,3,4,5,6,7]. The three naturally occurring CDs are composed of six, seven, or eight 1,4-linked glucose units for α-, β-, and γ-cyclodextrin, respectively (Figure 1 and Figure 2). Their three-dimensional structures have a truncated cone shape, with the primary (C_6_-OH) and secondary hydroxyl groups (C_2_- and C_3_-OH) lining the narrower and wider rim, respectively (Figure 1 and Figure 2). This confers some asymmetry and amphiphilicity to the CD structure, with the outside being more hydrophilic than the inside. The inside central cavity, whose size is determined by the number of glucose units forming the CD, can host various molecules to form stable inclusion complexes. The formation of these host–guest inclusion complexes typically involves the spatial entrapment of the guest molecule via intramolecular forces and does not involve the formation of covalent bonds [8].

Natural CDs and their derivatives (Figure 2) are widely used in pharmaceutical, cosmetic, and food products to improve solubility, increase stability, enhance bioavailability, convert liquids to powders, improve sensory qualities, reduce the likelihood of stomach irritation, inhibit hemolysis, etc. [6,7,10,11,12,13,14,15,16,17]. Because they contain multiple hydroxyl groups, CDs are water-soluble, but the water solubility of the natural CDs (α-, β-, and γ-cyclodextrin) is somewhat limited mainly due to internal hydrogen-bond formations. Because of this, derivatives such as (2-hydroxypropyl)-β-CD (HPβCD, hydroxypropyl betadex), sulfobutyl ether β-CD (SBEβCD), 2-hydroxypropyl-γCD (HPγCD), and others that have some of their hydroxyl groups substituted and are therefore more soluble are often used. Typically, these CDs are partially substituted at random sites on the parent molecules; thus, because of the large number of possible substitution sites (3 × 6, 3 × 7, or 3 × 8 hydroxyl groups for α-, β-, and γ-cyclodextrin, respectively), there are thousands of possible combinations [11]. Pharmocopeial monographs typically specify the degree of molar substitution (i.e., number of R groups per anhydroglucose unit, Figure 2); for example, HPβCD (hydroxypropyl betadex) should fall within the 0.4–1.5 range, meaning 2.8–10.5 hydroxypropyl groups per CD molecule (and it must be within 10% of the value stated on the label) [11].

The first cyclodextrin-based pharmaceutical was approved in Japan in 1976 (prostaglandin E_2_/βCD as Prostarmon E sublingual tablets), followed by Europe in 1988 (piroxicam/βCD as Brexin tablets), and the U.S. in 1997 (itraconazole/ HPβCD oral solution as Sporanox). Currently, over 100 drugs use CD complexes worldwide, along with various consumer products [6,7,10,11,12,13,14,15,16,17]. The clinical translatability of cyclodextrin complexes, in general, is further confirmed by numerous clinical trials ongoing with them—for recent up-to-date lists of products already approved or under investigation, see detailed reviews in [6,7,16,18]. The natural CDs (α-, β-, and γ-CD) are commonly used as food additives and are listed as GRAS (generally recognized as safe) by the FDA and as food additives by the WHO (World Health Organization). The EMA (European Medicines Agency) considers HPβCD and SBEβCD safe at relatively high doses in parenteral products, while it advises against using the natural αCD and βCD. Focusing on HPβCD, our main interest here, there are already several FDA-approved drug products that contain it, such as telavancin (marketed product: Vibativ; FDA approval: 2009), diclofenac sodium (Dyloject; 2014), letermovir (Prevymis; 2017), tecovirimat (Tpoxx; 2018), larotrectinib (Vitrakvi; 2018), and cladribine (Mavenclad; 2019) [19].

Here, after a brief discussion of a model relating molecular size and other physicochemical properties to CD complex stability, we review the use of CD complexes by our group (Bodor and coworkers) over several decades, highlighting their use to achieve clinical translatability for two cases: the development of oral tablets for cladribine by enhancing its bioavailability and the improvement of the solubility and stability of estredox, a brain-targeting chemical delivery system for estradiol.

## 2. CD Complex Stability

The stability of the host–guest complex depends on how well the guest can fit inside the cavity of the CD host. For example, we showed that the stability of the 1:1 complex (as measured by ln *K* or Δ*G*^0^) increases with the size of the guest molecule following a linear trendline up to a limit characteristic for each CD [8]. For guests larger than this limit, stabilities level off and are scattered around an average influenced by other properties such as shape, lipophilicity, and the presence of some specific structural elements (e.g., phenol moieties) (Figure 1). Notably, this type of behavior, including the values of corresponding slopes and intercepts obtained for α- and β-CD, was in excellent agreement with those predicted by the general framework of our unified, molecular size-based model for non-associative liquids [20].

The fit of the experimental values with a bilinear model such as LinBiExp [21] or a segmental linear regression (e.g., in GraphPad Prism) with an enforced horizontal second section gives slopes of –0.18 ± 0.02 and –0.16 ± 0.03 for β- and α-CD, respectively (where there were enough data points, *n* = 310 and 179; Figure 1), in excellent agreement with the model-derived slope of –0.20 for the size-dependency of the free energy of complexation, Δ*G*^0^ = –*RT*_0_ln*K* [8]. The fit for γ-CD, where there is also less data (*n* = 51), is less well defined, but both bilinear models gave a slope of –0.07 ± 0.01. The limiting size values obtained by the same fit (98 ± 5, 124 ± 3, and 282 ± 22 Å^3^ for α-, β-, and γ-CD, respectively) are also reasonable, as with the molecular volume calculations used for this model, they suggest cavity sizes that can accommodate around 6–7, 8–9, and 19–20 water molecules (*v*_water_ = 14.6 Å^3^). Estimates of the water molecules inside the cavity of natural CDs vary, but those based on experimental data are consistently around 6 and 10 for α- and β-CD, respectively, and much less certain, but possibly up to around 17 for γ-CD [8,22].

## 3. Improving Stability and Solubility: Estredox, a Brain-Targeting Retrometabolic Chemical Delivery System

The first application of CDs by our group (Bodor and coworkers) was in the second half of the 1980s to improve the formulation of chemical delivery systems (CDSs), which were introduced by the same group (Bodor and coworkers) a few years earlier (1981) to overcome the challenge of brain-targeted drug delivery imposed by the blood–brain barrier (BBB) [23]. The BBB, formed by non-fenestrated brain capillaries with tight junctions, restricts xenobiotic entry into the central nervous system (CNS). Entrance via passive diffusion is largely limited to lipid-soluble compounds; thus, poorly lipophilic therapeutics exhibit low brain penetration and need some delivery strategy to be able to reach the CNS.

### 3.1. Brain-Targeting Chemical Delivery Systems (CDSs)

CDSs are an important part of general retrometabolic drug design approaches—the corresponding principles and applications have been extensively reviewed [24,25]. CDSs are inactive chemical derivatives of drugs (D), generated via one or more structural modifications to enable site-specific or site-enhanced delivery through sequential enzymatic and/or chemical transformations. Originating from the prodrug concept [26,27], CDSs differ fundamentally by incorporating targetor (T) moieties and employing multistep activations. Unlike other brain-targeting strategies [28,29], CDSs aim to both enhance BBB influx and reduce efflux [24,25]. The CDS approach exploits the “lock-in” principle; the originally lipophilic molecule (T-D) crosses the BBB, then undergoes enzymatic conversion to a hydrophilic form (T^+^-D), preventing its return to circulation and providing sustained site-specific delivery of the active drug (T). As a targetor moiety, the 1,4-dihydrotrigonelline ⇄ trigonelline (coffearine) system proved particularly useful and has been employed for a wide variety of drugs [24,25]—the structure of estradiol-CDS (E_2_-CDS, estredox) is shown for illustration in Figure 2. The lipophilic dihydro form (T) is oxidized in vivo to a hydrophilic quaternary form (T^+^) via the ubiquitous NAD(P)H ⇄ NAD(P)^+^ coenzyme system, catalyzed by oxidoreductases involved in cellular respiration [30,31]. This transformation induces substantial changes in polarity, electron distribution, and lipophilicity, e.g., up to 4–5 orders of magnitude in log P values, thereby enabling efficient BBB penetration via the lipophilic T-D form and retention via lock-in of the hydrophilic T^+^-D form that can no longer cross back. Thus, retrometabolic CDSs are neither prodrugs nor pro-prodrugs as the correct sequential metabolism (T-D → T^+^-D → D), the BBB crossing and lock-in, and the systemic elimination of the hydrophilic precursor are all required.

### 3.2. CDS—Cyclodextrin Complexes

**Figure 3:**
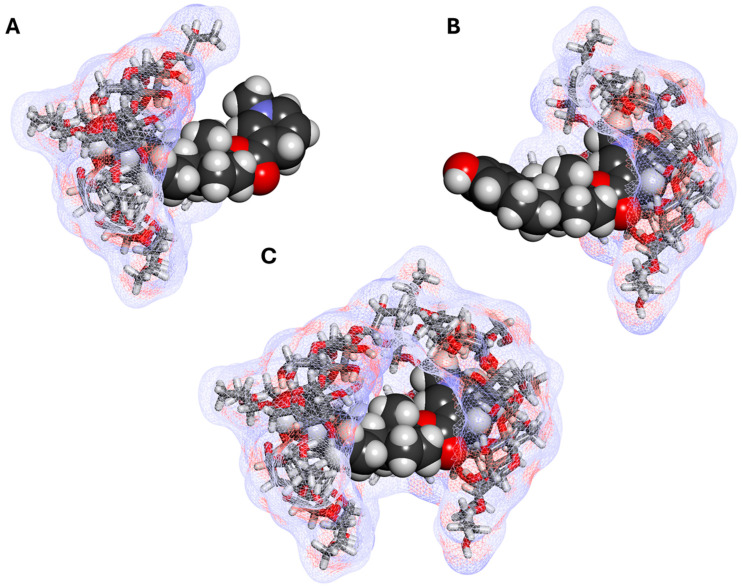
Possible estredox–HPβCD complexes: (**A**) a 1:1 complex with the steroid A ring in the CD cavity; (**B**) a 1:1 complex with the dihydropyridine ring of the targetor T moiety in the cavity; and (**C**) a 1:2 complex with both the A and T rings included. AM1-optimized structures [9], with estredox shown as a darker CPK (space filling) structure and the host CD molecules shown as lighter stick structures covered with a soft wire mesh surface colored by atomic charge using Biovia Discovery Studio Visualizer v25.

The physicochemical properties enabling effective CDS transport across the BBB often complicate pharmaceutical formulation. High lipophilicity facilitates deep brain penetration but results in poor aqueous solubility. Oxidative lability, essential for the lock-in mechanism, and hydrolytic sensitivity, required for drug release, limit CDS shelf life. Consequently, CDSs were formulated at first as solid powders for stability and administered via injection (after reconstitution) or transmucosal routes to bypass low oral bioavailability. Early animal studies used dimethyl sulfoxide (DMSO) for solubilization, but this was unsuitable for translation to human use. These challenges were overcome for the first time for testosterone–CDS in 1988 by employing HPβCD [32], a low-toxicity derivative [33] with enhanced water solubility due to disrupted hydrogen bonding that just became available at that time [34,35,36,37]. HPβCD then also led to significant progress in the formulation of estradiol–CDS (estredox) (Figure 2); it improved aqueous solubility ~250,000-fold (65.8 ng/mL to 16.36 mg/mL in 40% *w*/*v* HPβCD solution) and significantly increased oxidative stability, reducing ferricyanide-mediated oxidation rates tenfold (decreasing the rate constant *k* from 10.4 to 1.0 s^−1^M^−1^) and extending shelf-life fourfold (e.g., increasing the time required for 50% degradation *t*_50%_ at 60 °C from 20.4 to 76.2 days) [38]. Thus, HPβCD was selected due to its low toxicity and because it provided the best solubility enhancement among CDs tested (e.g., ~250,000-fold compared to 76-, 410-, and 334-fold for the natural α-, β-, and γ-cyclodextrins) [38]. Phase solubility analysis indicated 1:1 complex formation at low HPβCD concentrations and 1:2 complexes at higher levels (Figure 3). In addition to testosterone–CDS and E_2_-CDS, significant enhancements in solubility and stability were also achieved for other brain-targeting CDSs such as hydroxylomustine–CDS and benzylpenicillin–CDS. For example, benzylpenicillin–CDS’s aqueous solubility was enhanced about 70,000-fold (~60 ng/mL to 4.2 mg/mL in 20% *w*/*v* solution) and stability was also increased (e.g., the pseudo-first-order rate constant for overall loss in acidic media at pH 4.7 decreased more than ten-fold from 0.145 to 0.013 min^−1^) [39]. More detailed reviews of the use of CDs to improve the formulation of various retrometabolic CDSs can be found in [25,40].

### 3.3. Estredox—Clinical Applicability

Following the introduction of the HPβCD-based formulation, estredox achieved the most advanced development among retrometabolic CDSs so far by reaching Phase 2 clinical trials [25,40]. Although estrogens readily cross the BBB due to their lipophilicity, they are poorly retained in the CNS, necessitating frequent or sustained-release dosing—a shortcoming that estredox was designed to overcome. Brain-targeted estrogens that limit systemic exposure can have a variety of potential therapeutic applications including menopausal symptoms (“hot flashes”) [41,42], male and female sexual dysfunction [43,44,45], neuroprotection [46], depression [47,48], various types of dementia including Alzheimer’s disease [46,49,50], and others—see [25] for further details. Following its first synthesis in 1986 [51], estredox has been evaluated in multiple preclinical models, demonstrating selective and prolonged central effects, including the dose- and time-dependent suppression of luteinizing hormone (LH) and the modulation of copulatory behavior in ovariectomized female and castrated male rats. Comparative studies with conventional estrogen therapy further supported CNS selectivity. Four clinical trials of estredox demonstrated dose-dependent LH suppression in postmenopausal women without significant systemic estrogen elevation (see reviews in [25,40]). Buccal administration of estredox yielded potent, sustained CNS effects with minimal peripheral exposure, confirming therapeutic potential for safe treatment of menopausal symptoms, sexual dysfunction, and estrogen-dependent cognitive decline. Despite positive proof of CNS-targeted estradiol delivery and clinical efficacy, further development was discontinued due to budgetary constraints and intellectual property timing.

## 4. Improving Oral Bioavailability: Cladribine

A different, unrelated application of CDs by our group (Bodor and coworkers) is related to improving the oral bioavailability of cladribine [52,53]; it is reviewed for the first time here. Started in 2003, it ultimately resulted in a clinically approved product that received FDA approval in March 2019 (Mavenclad, oral cladribine tablet formulated with HPβCD) after extensive clinical trials demonstrated its effectiveness in reducing annualized relapse rates and the progression of disability in patients with relapsing-remitting and active secondary progressive multiple sclerosis (MS) [54,55,56,57,58].

### 4.1. Mavenclad—Oral Cladribine, a Disease Modifying Therapy for Relapsing Multiple Sclerosis (MS)

MS is a chronic, inflammatory, and neurodegenerative CNS disease, typically diagnosed in young adults aged 20 to 50. It is marked by frequent relapses, progressive disability, and cognitive decline. The disease is driven by autoreactive lymphocytes that trigger innate immune responses, also involving macrophages and leading to tissue damage, demyelination, and axonal injury characteristic of MS lesions. Cladribine (2-chlorodeoxyadenosine; Figure 2) is a synthetic purine nucleoside analog that selectively targets lymphocytes, resulting in the sustained depletion of circulating T and B cells implicated in MS pathogenesis [55]. It is a prodrug as its cytotoxicity is mediated by intracellular accumulation of phosphorylated cladribine in cells exhibiting a high deoxycytidine kinase-to-5′-nucleotidase ratio. These phosphorylated metabolites disrupt DNA synthesis and repair by incorporating into DNA and inhibiting key enzymes such as DNA polymerase and ribonucleotide reductase, ultimately inducing DNA strand breaks and apoptosis. Clinically, Mavenclad is administered as a short treatment course at the beginning of the first and second months of two consecutive treatment years. This relatively short duration of treatment cycles, combined with the convenience of oral administration, has made Mavenclad a valuable therapeutic option in MS, as other treatments typically require more frequent dosing or injectable administration. Mavenclad is now approved in >80 countries, and >100,000 patients have been treated with it, with Phase IV studies consistently demonstrating its safety and efficacy [59]. In 2023, it reached blockbuster status, achieving over USD 1 billion in annual sales [60].

Oral drug delivery is generally preferable over parenteral administration due to enhanced patient compliance and reduced healthcare costs [61,62,63]. Oral formulations minimize the need for clinical visits and mitigate the discomfort associated with injections or infusions; they also circumvent the expenses tied to the need for professional administration in clinical settings. However, the oral administration of cladribine was hampered by its limited oral bioavailability and pH sensitivity [64]. To overcome this, CD-based formulations were initiated, and a unique HPβCD-based formulation was developed [52,53].

### 4.2. Complex Dual Cladribine–Cyclodextrin Complex

Contrary to the traditional paradigm that used excess CD to enhance drug solubility with the assumption that this also protects acid-labile drugs from gastric degradation, our aim was to develop a saturated complex devoid of excess CD based on indications that surplus CD impairs cladribine absorption from solid oral and transmucosal dosage forms [52,53]. It was assumed that this formulation maintains cladribine at its highest thermodynamic activity upon contact with mucosal surfaces, thereby enhancing therapeutic efficacy. Along these lines, a formulation involving a unique dual cladribine- HPβCD complex was developed that is an amorphous admixture comprising an amorphous inclusion complex, where a hydrophobic moiety of cladribine is inserted into the CD cavity, and a non-inclusion complex, formed by hydrogen bonding between free amorphous cladribine and the exterior hydroxyl groups of the CD [53]. The formulation is optimized through controlled conditions involving elevated temperature, prolonged complexation time, and lyophilization to maximize cladribine incorporation while maintaining an amorphous state. Formation of this dual CD-complex is possible due to the sufficiently hydrophilic nature of cladribine, which has some aqueous solubility (4.5–5 mg/mL) [65], and the amorphous nature of the partially substituted HPβCD to achieve a supersaturated solution that, upon cooling, retains excess cladribine in a non-crystalline form. Typically, 60–70% of cladribine exists in the non-inclusion complex with the remainder in the traditional inclusion complex, though ratios can be adjusted by modifying the CD concentration and the processing conditions [53]. This dual-complex system uniquely enhanced drug solubility and allowed the formulation of oral tablets.

### 4.3. Phase Solubility Studies and Complex Structure

**Figure 4:**
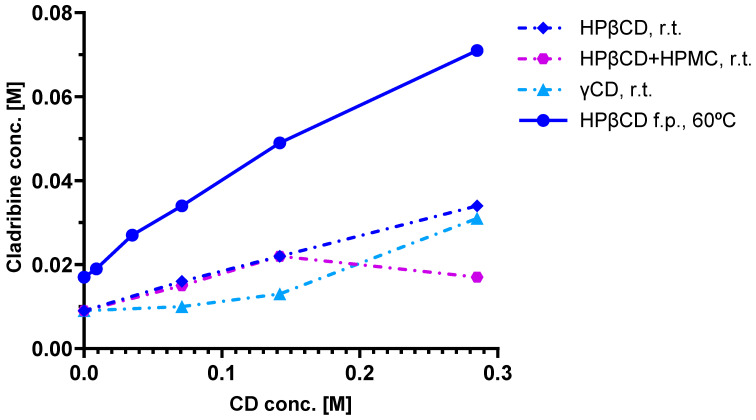
Results of phase solubility studies for cladribine–CD complexes at room temperature (25 °C, r.t.) and at 60 °C (fine particles, f.p.) [52,53]. CDs used as indicated in the legend, including a condition with added hydroxypropyl methylcellulose (HPMC); figure generated using GraphPad Prism version 10.5.

As a first step, phase solubility studies were carried out with HPβCD and γCD [52,53], which were chosen based on solubility and complex preparation considerations. Cladribine was added to CD solutions of various concentrations and allowed to complex; the excess undissolved cladribine was then removed by filtration, and the amount remaining in the complexation solution was measured. Figure 4 shows the phase solubility diagrams obtained for cladribine–CD complexes, including with HPβCD at two different conditions (room temperature and 60 °C). Linear curves such as those obtained for HPβCD indicate a 1:1 complex in which one guest molecule is complexed with one CD host. Note that in all of these complexes, additional CD is needed to maintain the cladribine in the complex; for example, even for the 60 °C condition with the highest cladribine content, around 0.15 mole of HPβCD is needed to maintain ~0.05 mole of cladribine dissolved (Figure 4).

**Figure 5:**
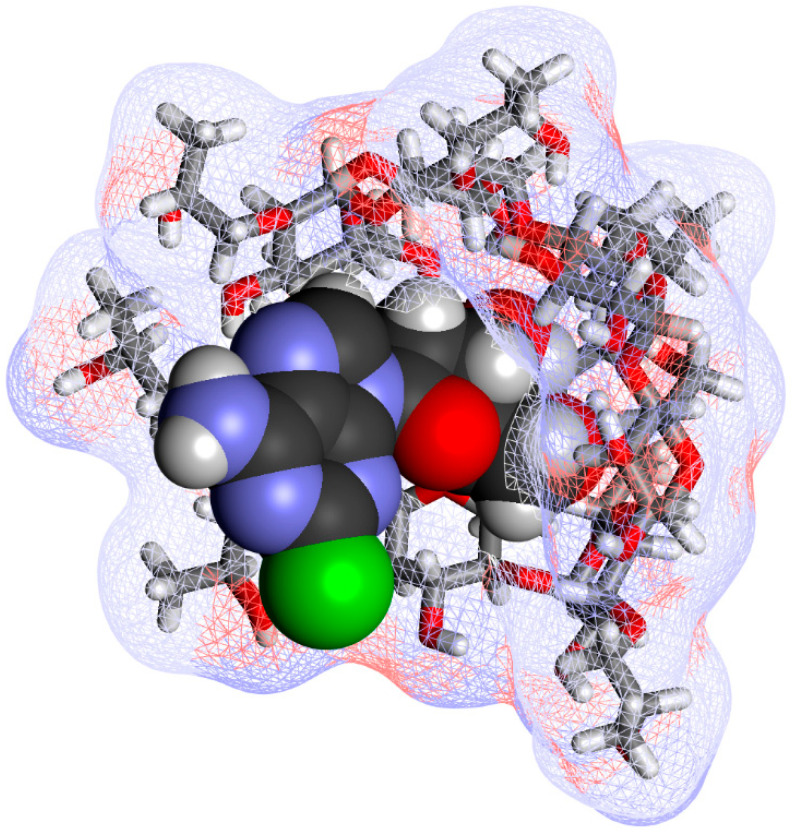
An illustrative structure of the cladribine-HPβCD complex structure. As in Figure 3, cladribine is shown as a darker CPK structure and the host HPβCD as a stick structure covered by a soft wire mesh surface using Biovia Discovery Studio Visualizer v25. The tetrahydrofuran ring of cladribine is included inside the HPβCD cavity, as suggested by the structural studies reported in [66].

Comprehensive chemical characterization of the inclusion complex has been carried out using thermal analyses (thermogravimetric analysis, TGA, and differential scanning calorimetry, DSC), vibrational analyses (both FT-IR and FT-Raman), and solid-state nuclear magnetic resonance (NMR), confirming the existence of an inclusion complex and showing that its characteristics are not affected by the manufacturing process and the complex is stable during storage [66]. A 2D ROESY experiment found cross-peaks between the inside protons at positions C_4_ and C_6_ of HPβCD and those at position C_4_ of the tetrahydrofuran ring of cladribine (Figure 2), indicating that cladribine and the inside lining of the CD cavity are in close proximity. A possible complex structure along these lines is shown for illustration in Figure 5.

### 4.4. Preclinical Pharmacokinetics

**Figure 6:**
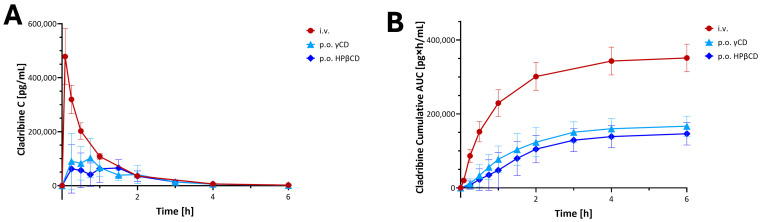
Preclinical PK of cladribine in a dog model [52]. Plasma concentration (**A**) and cumulative area under the concentration–time curve AUC (**B**) time-profiles for cladribine in dogs after administration of 5 mg single doses in the formulations as indicated. Data are average ± SD for *n* = 5–6 animals per group; figure generated using GraphPad Prism version 10.5. Only the first 6 h are shown of the 48 h test, as most concentrations returned to or near baseline by the end of this period.

Pharmacokinetic (PK) studies to evaluate the bioavailability of cladribine using these CD-based complexes were carried out in beagle dogs at IDRI (Dunakeszi, Hungary) [52]. Outbred male beagle dogs were allowed a laboratory diet and water ad libitum and were administered 5 mg cladribine in different formulations, including 0.25 mg/mL in isotonic saline intravenously (i.v.) and saturated cladribine–HPβCD or –γCD complex orally (p.o.). Serial blood samples were collected at various time intervals over 48 h. Cladribine levels in blood were measured by HPLC and LC/MS/MS. PK analysis was performed on individual plasma concentration versus time curves. Results are shown in Figure 6. Whereas the peak concentrations and the absorption profiles showed relatively high inter-individual variability after oral administrations, the total exposures (area under the concentration–time curve, AUC) showed much lower variability, and oral bioavailability was acceptable, being 44.8 ± 5.4% and 49.9 ± 2.8% for HPβCD and γ-CD, respectively [52]. These PK studies also confirmed that use of excess CD is counterproductive, and a saturated cladribine–CD complex substantially free of CD in excess of the minimum amount required to maintain substantially all the cladribine in the complex provided enhanced bioavailability as well as decreased intersubject variability. Based on the promising bioavailability data obtained in these studies, human studies were then initiated.

### 4.5. Clinical Pharmacokinetics

**Figure 7:**
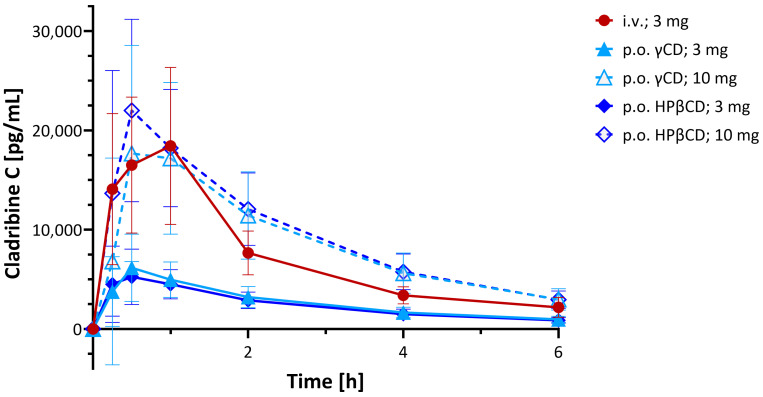
Clinical PK. Plasma concentration time profiles of cladribine after administration of two oral formulations (3.0 mg and 10.0 mg, p.o.) and one intravenous infusion (3.0 mg over one hour, i.v.) of single doses in the formulations as indicated [53,67]. Data are average ± SD for *n* = 26 MS patients; figure generated using GraphPad Prism version 10.5.

From the initial clinical studies performed, an open-label, randomized, three-way crossover study conducted across three centers to evaluate the systemic availability, safety, and tolerability of cladribine oral formulation in MS patients (IXR-102-09-186; IND No. 45,033) is summarized here [53,67]. Twenty-six participants received single doses of cladribine via two oral formulations (3.0 mg and 10.0 mg) and one intravenous infusion (3.0 mg over 1 h), with each administration separated by a minimum 5-day washout period. Plasma cladribine concentrations were quantified using LC/MS/MS for a 24 h sampling window. Results (Figure 7) indicated rapid absorption following oral administration with a median time to peak plasma concentration (*t*_max_) of ~0.5 h (range: 0.5–1.5 h) for the 10 mg dose. The mean maximum concentration (*C*_max_) ranged from 22 to 29 ng/mL, and the mean AUC ranged from 80 to 101 ng·h/mL, with results overall demonstrating dose-dependent PK and supporting the feasibility of oral cladribine delivery in MS therapy. There was no evidence of clinically important PK nonlinearity for the oral doses of 3 mg and 10 mg. This study concluded that the absolute bioavailability of the 3 mg and 10 mg oral formulations was approximately 34% and 39%, respectively, for the HPβCD tablets and 38% and 36% for the γ-CD ones (Figure 7) [53].

Based on these PK studies suggesting promising oral bioavailability in humans as well, efficacy studies were initiated. Because of the more favorable molar ratio as well as manufacturing considerations, the HPβCD complex was selected for further development. As mentioned, these subsequent clinical studies, including extensive Phase 3 and 4 clinical trials, have demonstrated the remarkable therapeutic efficacy of cladribine formulated with HPβCD as an oral tablet (Mavenclad) in patients with MS [54,55,56,57,58]. Due to the convenience of oral administration and relatively short treatment cycles, it has now reached blockbuster status by achieving >USD 1 billion in annual sales [60].

## 5. Conclusions

The work summarized here highlights the usefulness of CD complexes and especially HPβCD complexes for clinical translatability. They improved the solubility and stability of retrometabolic CDSs, in general, and estredox (E_2_-CDS) in particular, and they made the development of oral tablets for cladribine possible by enhancing bioavailability. Complexation with HPβCD dramatically increased the water solubility and oxidative stability of estredox, making its translation to clinical development possible. Results of these clinical trials highlighted the potential of our brain-targeted CDS approach for achieving a CNS-targeted effect with minimal peripheral side effects. An HPβCD-based formulation also provided an innovative solution for the development of oral cladribine tablets by ensuring formulability and adequate bioavailability, as demonstrated in both preclinical and clinical studies, which were briefly reviewed. The resulting Mavenclad tablets offer convenient, effective, and well-tolerated oral therapy for MS, achieving worldwide approval and significant clinical success. These works initiated in our laboratories over a period of more than three decades underscore the potential of CD-based approaches for overcoming barriers in drug formulations, especially for compounds with challenging physicochemical properties. As, in addition to those discussed here, there are multiple other HPβCD-containing drug products in development or in clinical trials [6,7,18], it is certain that the list of those already approved for clinical use [16,19] will continue to grow.

## Data Availability

No new data were created or analyzed in this study. Data sharing is not applicable to this article.

## Abbreviations

The following abbreviations are used in this manuscript:

AUC	Area under the concentration–time curve
BBB	Blood–brain barrier
CD	Cyclodextrin
CDS	Chemical delivery system
CNS	Central nervous system
CPK	Corey–Pauling–Koltun (model)
HPβCD	(2-Hydroxypropyl)-beta cyclodextrin; hydroxypropyl betadex
MS	Multiple sclerosis
PK	Pharmacokinetic
